# Transient co-assemblies of micron-scale colloids regulated by ATP-fueled reaction networks†

**DOI:** 10.1039/d3sc04017h

**Published:** 2023-10-16

**Authors:** Charu Sharma, Aritra Sarkar, Andreas Walther

**Affiliations:** a Department of Chemistry, Life-Like Materials and Systems, University of Mainz Duesbergweg 10-14 55128 Mainz Germany andreas.walther@uni-mainz.de

## Abstract

Self-assembly of colloidal particles offers an attractive bottom-up approach to functional materials. Current design strategies for colloidal assemblies are mostly based on thermodynamically controlled principles and lack autonomous behavior. The next advance in the properties of colloidal assemblies will come from coupling these structures to out-of-equilibrium chemical reaction networks furnishing them with autonomous and dynamic behavior. This, however, constitutes a major challenge of carefully modulating the interparticle potentials on a temporal circuit program and avoiding kinetic trapping and irreversible aggregation. Herein, we report the coupling of a fuel-driven DNA-based enzymatic reaction network (ERN) to micron-sized colloidal particles to achieve their transient co-assembly. The ERN operating on the molecular level transiently releases an Output strand which links two DNA functionalized microgel particles together into co-assemblies with a programmable assembly lifetime. The system generates minimal waste and recovers all components of the ERN after the consumption of the ATP fuel. The system can be reactivated by addition of new fuel as shown for up to three cycles. The design can be applied to organize other building blocks into hierarchical structures and materials with advanced biomimetic properties.

## Abstract 2

An ATP-fueled DNA based enzymatic reaction network regulates transient co-assemblies of micron-scale colloids. The system produces minimal waste, shows ATP-dependent behavior, and can be reactivated for up to three consecutive cycles.

## Graphical abstract

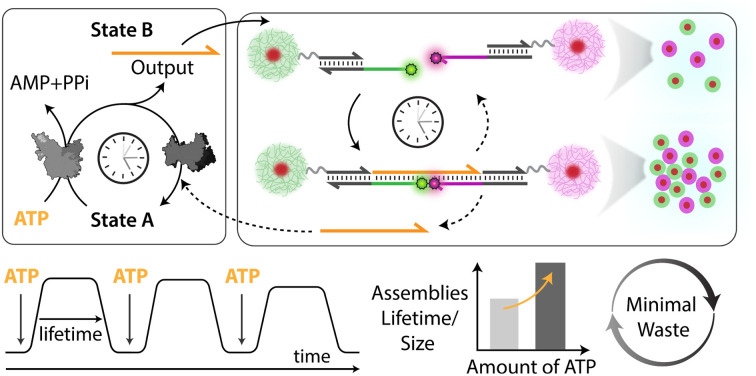

## Introduction

Self-assembly of synthetic building blocks (molecular or colloidal) is an extremely powerful tool for the development of complex systems and materials in a bottom-up fashion. The past decade has experienced a gradual evolution in their properties from being classic responsive to adaptive and life-like in nature.^1–3^ On a conceptual level, such systems often take inspiration from biological self-assemblies such as microtubules and actin filaments which operate out of equilibrium *via* hierarchically concatenated chemical reaction networks (CRNs) and regulate advanced cellular functions including cell-division, motility, transport, and proliferation.^4^ Being able to translate such behavior into present-day synthetic self-assemblies remains a challenging goal to create dynamic and adaptive materials unachievable by conventional equilibrium principles. This can be achieved by either coupling the responsive building blocks to energy-dissipating environments or designing the building blocks to be energy dissipating themselves.^5^ The first strategy provides a more generic approach, whereas the latter one typically requires judicious design and tuning of the CRN for each building block.

Choosing appropriate synthetic analogues to emulate biological self-assemblies is essential to obtain emergent life-like behavior. This has been demonstrated for various building blocks from different chemical origins and domains such as small molecules,^6–11^ colloids,^11–19^ DNA,^20–23^ peptides,^18,24,25^ surfactants^26^ up to molecular machines.^27,28^ Colloids especially can give rise to functional materials with unique catalytic, photonic, magnetic, and electronic properties.^29–31^ These properties originate not only from the positional or dimensional order of these building blocks, but also from the type of material bulk (metallic, polymeric, semiconductor, inorganic). Employing biocompatible materials for their fabrication can even pave the way into biomedical applications such as drug delivery and diagnostics.^32^

Imprinting transient behavior into colloidal particles requires precise control over the interparticle potentials such that the repulsive contributions (steric/electrostatic) dominate the attractive van der Waals interactions in the deactivated or disassembled state of the particles. A slight disbalance can otherwise compromise the overall stability of the particles and result in irreversible aggregation. Only in the activated state should the fuel-induced attractions sufficiently overcome repulsion to induce assembly. Limiting the magnitude of attractions between the particles by either reducing the binding strengths or the number of activated sites is essential to avoid the system falling into kinetic traps and promote spontaneous disintegration of assemblies after fuel depletion.^14,33^ Another challenge involves fine tuning of a fuel-consuming CRN in terms of its activation and deactivation rates to match the colloidal dynamics which are significantly slower than their molecular counterparts. Naturally, the challenges become more evident when larger colloids are used. Because of which the majority of transient colloidal systems demonstrated till date rely on relatively small (*z*-average diameter, *D*_*z*_ ≈ 5–50 nm),^1^ nanoparticle-sized building blocks while examples for micrometer-sized particles are scarce.^13,15–17^

For instance, van Ravensteijn and Hendriksen *et al.* demonstrated the first example for the transient clustering of sub micrometer-sized (*D*_*z*_ ≈ 750 nm) colloidal particles by modulating the hydrophobicity of the grafted polymers in response to a limited supply of chemical fuel.^15^ The system, however, lacks selectivity and cannot be utilized further for multicomponent systems. We recently coupled pH-feedback system to pH-responsive microparticles to achieve transient co-assembling systems.^13^ The excessive salt accumulation in these systems can disbalance the interparticle potentials and thereby the colloidal stability after each fueling cycle. In this respect, DNA coated colloids may offer better structural control due to high programmability and selectivity of DNA hybridization.^16,17^ Particularly this allows design of DNA-based out-of-equilibrium CRNs emulating complex signaling networks found in nature. In this direction, DNA-based CRNs coupled to DNA-coated particles with transient and oscillatory behavior were reported. Dehne *et al.* showed transient assembly of DNA-coated colloids (*D*_*z*_ ≈ 1 μm) using antagonistic enzymatic CRN of RNA formation and degradation.^17^ They also programmed oscillation of these colloids by coupling them to polymerase, exonuclease and nickase (PEN)-based enzymatic CRNs.^16^ In both cases, the transiently generated linker strand is based on nucleotide monomers that are degraded to waste. This equals an energetically downhill process opposed to what is found in nature where a chemical fuel is processed and the building components themselves are not degraded. We recently integrated a toehold-mediated DNA strand displacement reaction network with micron-sized colloids and DNA origami nanoparticles to regulate the transient co-assembly and self-sorting of two building blocks.^34,35^ Those systems however reach new thermodynamic states at the end and cannot be refueled. Therefore, examples where fuel-driven DNA-based out-of-equilibrium reaction networks can be utilized for temporal and spatial control over DNA-coated colloidal co-assemblies remain very scarce.

Over the past few years, we have developed ATP-driven enzymatic reaction network (ERN) to achieve dynamic behavior in systems ranging from polymer level^21,36,37^ to nanostructures^38^ up to multicomponent self-sorted colloids (*D*_*z*_ ≈ 1 μm) and coacervates.^19,39^ Herein, we build on our ATP-mediated dissipative strand displacement cascade whereby a linker strand is made transiently available in the system by ATP-powered ligation and restriction of DNA components to operate a temporally controlled downstream co-assembly of larger micron-sized core–shell microgels (MGs). The linker strand is recovered after the consumption of the ATP fuel allowing it to be reused for subsequent cycles. We discuss in detail how to modulate such ERNs, how to fabricate and couple particles to these ERNs to exclusively achieve non-equilibrium structures avoiding irreversible aggregation and kinetic traps. Our strategy offers design principles for an unattended dimension of particles and can practically be adopted for building blocks of different chemical origin and varied dimensions. We envisage that upon appropriate modification of colloids or materials, their interactions with cells can be programmed in a temporal fashion in their co-assembled state.^40^

## Results and discussion

### System design for transient colloidal co-assemblies using ATP-fueled ERN

**Fig. 1:**
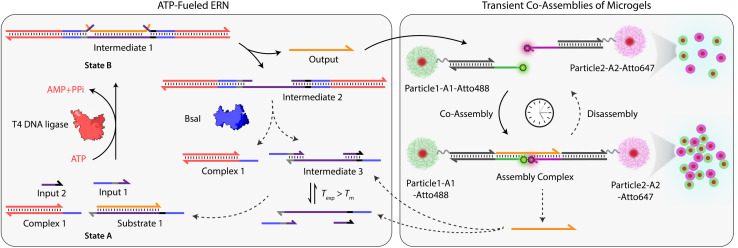
System design for ATP-driven transient co-assemblies of MGs regulated by upstream ATP-fueled ERN.

Building on our previous design, we customized ATP-fueled ERNs of antagonistic ATP-powered ligation and restriction for an easy concatenation with colloidal particles (Fig. 1). The ATP-fueled ERN operates on the molecular level and functions as a control program for the autonomous co-assembly of particles at the structural level. The upstream ERN in its initial deactivated State A is composed of two double-stranded (ds) complexes (Complex 1 and Substrate 1) and two single stranded DNAs (ssDNA; Input 1 and Input 2) as shown in Fig. 1. In this state, Input 1 and Input 2 are incapable of kicking out Output from Substrate 1 due to unstable hybridization of Input 1 (melting temperature (*T*_m_) = 32 °C < experimental temperature (*T*_exp_) = 37 °C, determined by NUPACK^41^) and Input 2 (*T*_m_ = 22 °C < *T*_exp_ = 37 °C) with the longer strand of Substrate 1. However, the addition of ATP powers the covalent ligation of Substrate 1 with two molecules of Complex 1 (4 equivalents (equiv.) used with respect to Substrate 1) and one molecule each of Input 1 and Input 2 with the help of T4 DNA ligase, generating Intermediate 1. The system is pushed towards an activated State B, where the chain migration from two sides provides a strong thermodynamic push to release Output from Intermediate 1, finally generating Intermediate 2. The dual inversion strategy is essential for releasing an Output strand long enough to link two particles together in a ternary Assembly Complex (*vide infra*).^37,38^ Concurrently, BsaI cleaves Intermediate 2 to regenerate Complex 1 and Intermediate 3. Because of the low *T*_m_ (32 °C < *T*_exp_ = 37 °C), Intermediate 3 dissociates into Input 1 and Input 2 and reproduces Substrate 1 after re-hybridization with Output returning the system back to State A. Faster ligation than cleavage (ESI Note 4.1†) satisfies the kinetic boundary conditions required for the system to achieve a dynamic steady state with an ATP-dependent lifetime before the restriction overtakes and brings the system back to its initial state. Output is therefore generated in an uphill fashion and is transiently available in the system to perform downstream functions before it is ultimately reassociated within Substrate 1. The co-assemblies are thereby maintained by an ATP-driven dynamic steady state where the lifetime and the size of the hierarchical structures can be programmed by the amount of ATP introduced in the system.

### Characterization of the ATP-fueled ERN

**Fig. 2:**
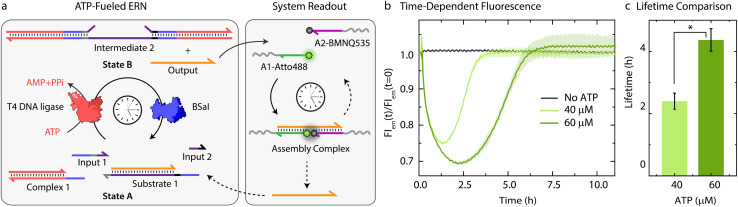
*In situ* detection of transiently released Output strand from an ATP-fueled DNA-based ERN. (a) Schematic representation for the *in situ* readout of transiently released Output strand *via* FRET interaction between A1-Atto488 and A2-BMNQ535. (b) Time-dependent FI changes demonstrating programmable transient complexation between A1-Atto488, A2-BMNQ535, and Output with different ATP equivalent. (c) Corresponding lifetime obtained from (b) shows an increase with higher ATP equivalent. Experimental conditions: A1-Atto488 and A2-BMNQ535 at an equimolar concentration of 5 μM are dissolved in 1× NEB CutSmart buffer containing 20 μM Complex 1, 5 μM Substrate 1, 10 μM Input 1 and Input 2 at 37 °C, initiated by different ATP concentrations. The results represent an average contribution from two measurements, the shaded region in (b) and error bars in (c) depict the standard deviation (SD). * indicate that *p* < 0.05.

Before coupling the ATP-fueled ERN to particles, we first sought to construct the ATP-driven ERN in solution (Fig. 2a). The system works as follows: fluorophore-functionalized A1-Atto488 and quencher-functionalized A2-BMNQ535 which will be later annealed on the particles are dissolved in solution together with the other components of the upstream ERN. The released Output strand, powered by ATP-fueled ligation, will couple A1-Atto488 and A2-BMNQ535 into an Assembly Complex, resulting in a decrease in fluorescence intensity (FI) due to close proximity between the fluorophore and quencher. Subsequently, once BsaI controlled restriction takes over, the Assembly Complex will be dissociated resulting in the recovery of the fluorescence signal. For the successful functioning of the system at both upstream and downstream nodes, two conditions are mandatory: (1) in the initial State A, Output must remain bound within Substrate 1 without showing crosstalk with A1-Atto488 and A2-BMNQ535. This condition is also essential for the system to regenerate Substrate 1 and return back to State A after consumption of ATP. Overall, the hybridization of Output within Substrate 1 should be more favorable than formation of the Assembly Complex with A1-Atto488 and A2-BMNQ535 in State A; (2) in State B (ATP-fueled), binding of Output with A1-Atto488 and A2-BMNQ535 must be more favorable than its tendency to remain bound within Intermediate 1. For both of these conditions to be met, the length and composition of Output play critical roles. For instance, a longer Output strand with higher binding affinity is essential to drive the ternary Assembly Complex formation between A1-Atto488 and A2-BMNQ535, and Output. On the other hand, the increased length of the Output can pose difficulty during its expulsion from Intermediate 1. As a trade-off, we added 2 nucleotides (nt) at the 3′-end of Output (ESI Table S2†) which are non-complementary to the long strand of Substrate 1 and hence, do not affect the release of Output from Intermediate 1. However, this 2 nt extension successfully induces the formation of the Assembly Complex by strengthening the binding of Output with A1-Atto488 and A2-BMNQ535 (Fig. S1†).

Next, with the designed strands we assembled the ERN in solution at 37 °C with 20 μM Complex 1, 5 μM Substrate 1, 5 μM A1-Atto488 and A2-Atto647, 10 μM Input 1 and Input 2 using 0.8 Weiss units (WU) μL^−1^ of T4 DNA ligase and 0.8 units (U) μL^−1^ of BsaI. The ERN was initiated by ATP addition, which immediately resulted in a decrease in fluorescence signifying the formation of the Assembly Complex between A1-Atto488, A2-BMNQ535 and Output. Once ATP reaches a subcritical level, the restriction of Intermediate 2 *via* BsaI overtakes ligation and leads to regeneration of Substrate 1 returning the system back to State A with the full recovery of FI. The time-dependent FI measurements confirm the ATP-powered ERN with transient population of the Assembly Complex between A1-Atto488, A2-BMNQ535 and Output (Fig. 2b). No such change in the FI is observed in the absence of ATP. The lifetime of the ERN reaction can be further modulated using a higher ATP concentrations which allow ligation to dominate restriction process for longer period.^42^ Because of this the dynamic steady state is (a) even more populated with Output strand thereby increasing the maximum dynamic yield of the Assembly Complex which can be explained by the 32% decrease in FI at 60 μM ATP as opposed to 25% decrease in case of 40 μM ATP (Fig. 2b), and (b) acquired later which increases the lifetimes of the Assembly Complex from ∼2.4 to ∼4.3 h (Fig. 2c). Through control experiments, we found that mixing 5 μM each of A1-Atto488, A2-BMNQ535 and Output generates 2.6 μM (52%) of Assembly Complex in equilibrium (Fig. S2b and c†). NUPACK simulation suggests a similar yield of 54.8% (Fig. S2a†). Hence, we can conclude that fueling the system with 40 μM ATP furnishes ∼1.16 μM (23%) of Assembly Complex (check Fig. S3† for detailed explanation). Similarly, for 60 μM ATP, ∼1.48 μM (29%) of Assembly Complex is formed.

An interesting calculation regarding the question on how often building blocks are reactivated can be made. Substrate 1 is present at 5 μM and requires 4 ligation steps (Fig. 1) that will consume 4 ATP molecules to eject one Output strand with highest efficiency. That means that each Output can at maximum be transduced 3 times in the cyclic ERN at 60 μM ATP. In reality, this will be lower as the restriction can already set in at the hemi-ligated intermediate (*e.g.*, Substrate 1, Complex 1 and only one of the Inputs) without completing to the fully ligated state that is the condition for expulsion of Output (Fig. S3†).

The system can be successfully reactivated up to 3 times (Fig. S4†). However, we noted that for subsequent cycles of ATP addition, the decrease and increase of FI is slowed down, increasing the lifetime of the Assembly Complex by more than 3 times (6th cycle) compared to the previous cycles. This might occur due to gradual loss of enzymes activities over time.^21,37,43^

### DNA-functionalized MGs and their hetero-complementary co-assembly

**Fig. 3:**
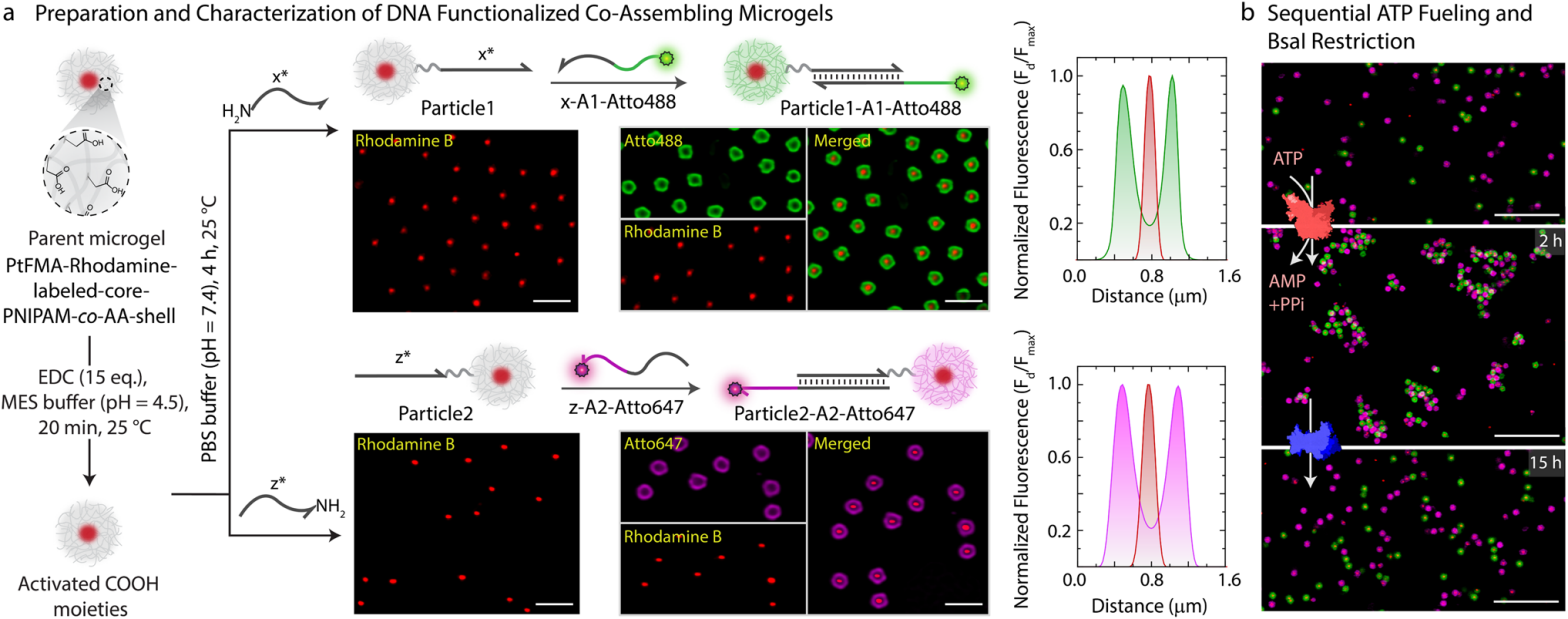
Fabrication and characterization of DNA-functionalized co-assembling MGs. (a) Schematic and CLSM images depicting step-wise preparation of Particle1-A1-Atto488 and Particle2-A2-Atto647 from a parent microgel. The first step involves covalent functionalization of DNA and consequent annealing with *x*-A1-Atto488 on Particle1 and *z*-A2-Atto647 on Particle2. Each step is monitored with CLSM imaging, and the particles are quantitatively analyzed by line segment analysis. Experimental conditions: Particle1 and Particle2 suspended in TE buffer (pH = 8.0) at a final concentration of 0.05 wt%, to which 0.63 μM (1 equiv. with respect to functionalized DNA) of respective annealing strands are added at 15 °C. (b) CLSM images of sequential assembly and disassembly of Particle1-A1-Atto488 and Particle2-A2-Atto647 using ATP-driven ligation and disassembly using subsequent addition of BsaI. The sample was first checked for co-assembly after 2 h of 40 μM ATP addition with *ex situ* CLSM imaging. After addition of BsaI strand, sample was again visualized after 15 h. Experimental conditions: Particle1-A1-Atto488 and Particle2-A2-Atto647 suspended in 1× NEB CutSmart buffer at a final MG concentration of 0.05 wt% with 5 μM Substrate 1, 20 μM Complex 1, 10 μM Input 1, 10 μM Input 2, 0.8 WU μL^−1^ T4 DNA ligase followed by addition of 0.8 U μL^−1^ of BsaI at 37 °C. All CLSM images in b are represented as merged composite compiled as a *z*-projection. Scale bars: (a) 2 μm, (b) 10 μm.

With successful demonstration of the ATP-powered ERN and transient formation of Assembly Complex between A1-Atto488, A2-BMNQ535, and Output, we next attempted the translation to transient co-assemblies between DNA-functionalized core–shell MGs. For this, we employ DNA-functionalized micron-sized core–shell MGs as building blocks as they are well dispersible in aqueous medium and can be functionalized within their hydrogel shell.^44,45^ The core is composed of a brightly red fluorescent hydrophobic material, refractive index-matched to water (〈*R*_h_〉_*z*_ ≈ 230 nm, synthesis in ESI Section 3.1†). The shell contains a lightly crosslinked PNIPAM-*co*-AA hydrogel constituting 90.5 wt% of the MG (80.5 wt% *N*-isopropylacrylamide (NIPAM); 9 wt% acrylic acid (AA); 1 wt% *N*,*N*′-methylenebis(acrylamide) (MBA)). The core–shell MGs have a total 〈*R*_h_〉_*z*_ ≈ 1.4 μm and can be well visualized in Confocal Laser Scanning Microscopy (CLSM, Figs. 3 and S5†). The AA groups in the shell allow functionalization with NH_2_-ssDNA (Fig. 3a, synthesis in ESI Section 3.2†). This can be achieved by EDC-mediated coupling of NH_2_-ssDNA (*e.g.*, NH_2_-x* and NH_2_-z*; EDC = 1-ethyl-3-(3-dimethylaminopropyl)carbodiimide = 25 equiv. with respect to COOH groups on MG), leading to a typical DNA grafting density of 2.7 × 10^3^ strands per MG or 1.26 ± 0.2 μmol strands per g of MG (ESI Section 3.3, Fig. S6†). Such grafting densities agree with previous literature for DNA-mediated colloidal assembly.^46,47^ By using two different NH_2_-ssDNA, we prepared an x*-functionalized Particle1 and a z*-functionalized Particle2. Subsequently, we annealed the potential binding components on each of the particles. Particle1 at a final concentration of 0.05 wt% was incubated with equimolar amount of complementary *x*-A1-Atto488 (0.63 μM). Similarly, Particle2 at a final concentration of 0.05 wt% was incubated with equimolar amount of *z*-A2-Atto647 (0.63 μM). Please note that the concentration of DNA is comparable to the concentration of Assembly Complex (Fig. 2b) which is formed in the transient state. The post-annealing with the dye labeled strands (*x*-A1-Atto488, and *z*-A2-Atto647) offers the advantage of visualizing successful functionalization by CLSM as both Particle1-A1-Atto488 and Particle2-A2-Atto647 turn fluorescent with orthogonal emission profiles (Fig. 3a).

We hypothesized that the transient release of Output from the upstream ATP-fueled ERN should bring Particle1-A1-Atto488 and Particle2-A2-Atto647 together into the co-assemblies. But for consistency and setting a reference, we first investigated the sequential co-assembly using T4 DNA ligase and disassembly using BsaI.

The system contains an equimolar mixture of Particle1-A1-Atto488 and Particle2-A2-Atto647 at a final concentration of 0.05 wt% dispersed in medium containing all the DNA components of the ERN, 20 μM Complex 1, 5 μM Substrate 1, 10 μM Input 1 and Input 2 and 0.8 WU μL^−1^ of T4 DNA ligase. CLSM images confirm the co-assemblies between the particles after 2 h of introducing 40 μM of ATP in the absence of the restriction enzyme BsaI (Fig. 3b). The co-assemblies achieve an average size of 9 μm^2^ as confirmed by automated image analysis (Fig. S7†). Subsequent introduction of 0.8 U μL^−1^ of BsaI into the system recovers the Output strand from the formed structures and induces disassembly into individual particles after 15 h.

### ATP-fueled autonomous co-assemblies of MGs

**Fig. 4:**
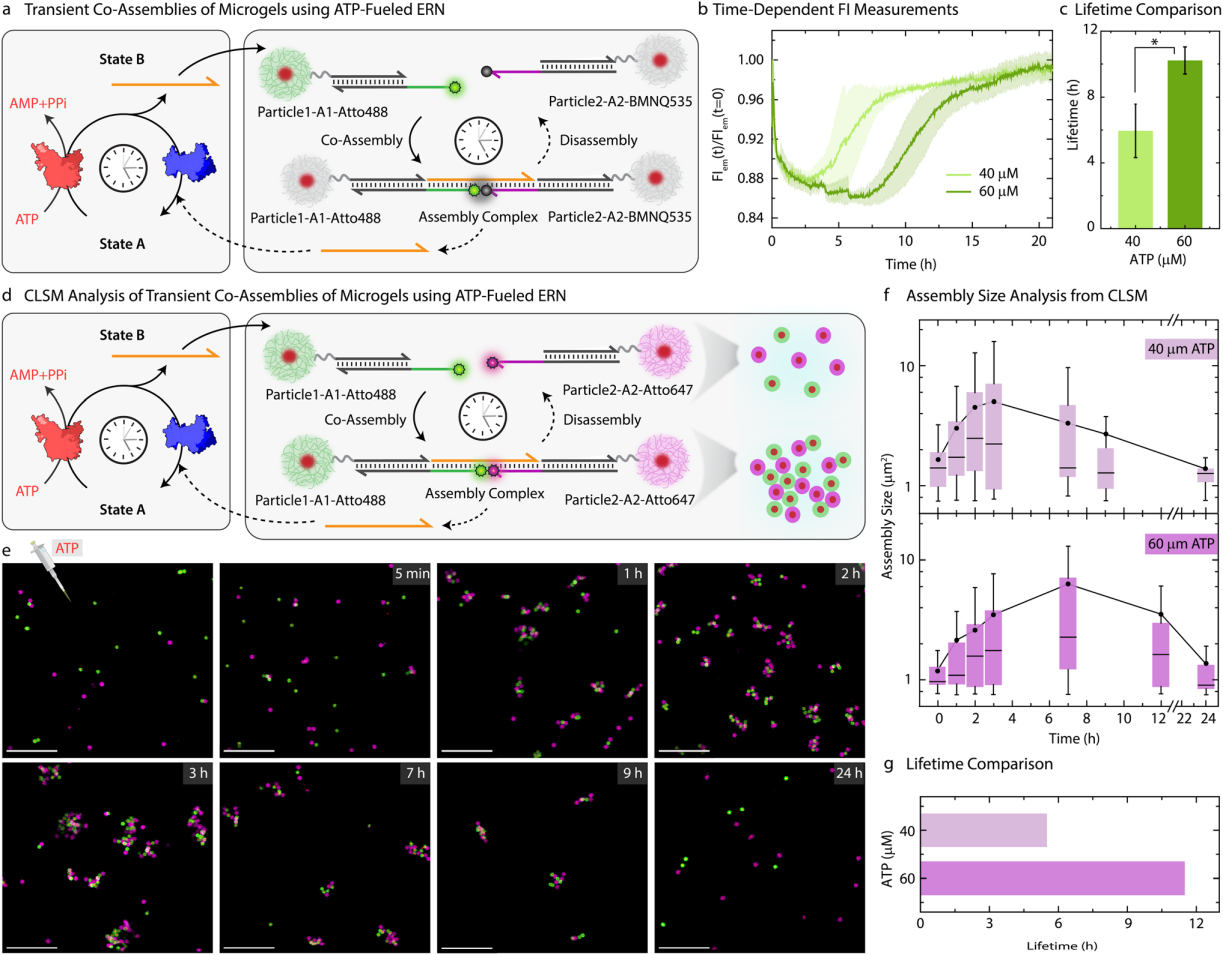
Self-regulating co-assembling MGs with transient lifetimes. (a) Schematic representation showing transient co-assembly between Particle1-A1-Atto488 and Particle2-A2-BMNQ535 due to transiently released Output strand from ATP driven enzymatic reaction network. (b) Time-dependent FI changes demonstrating transient co-assembly of dye functionalized Particle1-A1-Atto488 and quencher functionalized Particle2-A2-BMNQ535 upon ATP addition (c) lifetime comparison of the ATP driven transient co-assembly of Particle1-A1-Atto488 and Particle2-A2-BMNQ535 with different ATP equivalent demonstrating an increase in lifetime with increase in ATP equivalent. Experimental conditions: Particle1-A1-Atto488 and Particle2-A2-BMNQ535 are suspended as an equimolar mixture in 1× NEB CutSmart buffer at a final MG concentration of 0.05 wt% containing 20 μM Complex 1, 5 μM Substrate 1, 10 μM Input 1 and Input 2 at 37 °C, fueled by different ATP concentrations. The results represent an average contribution from two measurements, the shaded region in (b) and error bars in (c) depict the SD. * indicate that *p* < 0.05. (d) Schematic representation for CLSM analysis of the transient co-assemblies between Particle1-A1-Atto488 and Particle2-A2-Atto647. (e) *Ex situ* CLSM imaging of transient co-assemblies at 40 μM ATP concentration. All CLSM images are represented as merged composite compiled as a *z*-projection. Experimental conditions: same as described in (b) except using Particle2-A2-Atto647 instead of Particle2-BMNQ535. Aliquots were withdrawn as needed and visualized without any dilution. (f) Assembly size analysis on the particles obtained from two different *z*-stacks at each time interval observed at two different ATP concentrations. The box represents 25–75% of data, whiskers represent 5–95% of data, a solid circle represents the mean, and horizontal bar the median of the assembly size distribution in box charts. A solid line provides a guide to the eye. (g) ATP-dependent lifetime of the transient co-assemblies obtained from the assembly size analysis. Scale bars: 10 μm.

Moving beyond the classical step-by-step switching by sequential addition of ligation and restriction components, we next targeted the ATP-fueled ERN with Particle1-A1-Atto488 and Particle2-A2-Atto647 to achieve transient self-regulating co-assemblies (Fig. 4a).

We decided to probe the co-assemblies of the particles using both time-dependent FI changes as well as CLSM. To monitor the transient co-assemblies using time-dependent FI changes we annealed Particle2 with *z*-A2-BMNQ535. The presence of a quencher on Particle2-A2-BMNQ535 will *in situ* report any formation of co-assemblies between Particle1-A1-Atto488 and Particle2-A2-BMNQ535 owing to the FRET mechanism. With the fluorophore and quencher modified particles in hand, we constructed the ATP-fueled ERN by combining 20 μM Complex 1, 5 μM Substrate 1, 10 μM Input 1 and Input 2, 0.8 WU μL^−1^ of T4 DNA ligase and 0.8 units (U) μL^−1^ of BsaI containing an equimolar mixture of Particle1-A1-Atto488 and Particle2-A2-BMNQ535. Before adding ATP, the system shows high FI because there are no interactions between the two particles (Fig. 4b). However, the addition of ATP activates the ligation-induced transient release of Output which links Particle1-A1-Atto488 and Particle2-A2-BMNQ535 together to quench Atto488 fluorescence due to the formation of the Assembly Complex. A FI decrease of ∼12% occurs. Once ATP is consumed, the restriction reaction dominates, leading to the disassembly of the transiently formed co-assemblies marked by the complete recovery of the FI. The linker-mediated transient co-assembly between the particles can be further temporally controlled by increasing the concentration of ATP from 40 to 60 μM, which leads to an increase in the lifetime from 6 to 10 h (Fig. 4c). Much higher ATP concentrations lead to more pronounced decrease in FI suggesting an increased yield of Assembly Complex with an ATP-dependent lifetime (Fig. S8†).

Moreover, a variation in the enzyme concentration also changes the lifetime and yield of the Assembly complex. Towards this, we used a two-fold excess of T4 DNA ligase (1.6 WU μL^−1^) as compared to BsaI (0.8 U μL^−1^), while keeping the concentrations of DNA species and ATP fixed (Fig. S9†). This leads to two main effects: (1) a faster and greater decrease in FI (32%) compared to the original system (12%) indicates a faster establishment of dynamic steady state with an increased fraction of Assembly Complex; (2) a shorter lifetime of 2.4 h as compared to standard system (1 : 1 ratio of T4 DNA ligase and Bsai, lifetime = 6.4 h) indicates a faster ATP consumption during ligation allowing restriction to overtake ligation much earlier. However, no transient state is observed in the absence of BsaI. A parallel experiment with two-fold excess units of BsaI (1.6 U μL^−1^) as compared to T4 DNA ligase (0.8 WU μL^−1^) at fixed concentrations of DNA species and ATP favors restriction more than ligation. More frequent cleavage events lead to decrease in both the lifetime (1.9 h) as well as the yield of the Assembly Complex. This is evident from a relatively less decrease in FI on ATP addition (9%) compared to pristine system with 1 : 1 ratio of the BsaI and T4 DNA ligase.

Since the particles can be easily labeled with fluorophore-conjugated DNA species, the transient co-assembly can be convincingly visualized using CLSM. For this, we annealed *z*-A2-Atto647 on the Particle2 instead of using Particle2-A2-BMNQ (Fig. 4d and e). The assembled ERN/MG mixture prior to ATP addition showed well dispersed green and pink MGs (Fig. S10†). However, within 1 h of ATP addition, small co-assembled clusters are observed which eventually grow and reach a steady plateau (average assembly size ≈ 5 μm^2^) after 3 h beyond which they begin to disassemble into individual MGs when observed until 24 h (Fig. 4f) confirmed by automated image analysis. This clearly suggests transient formation of co-assemblies. However, for a higher ATP concentration, the co-assemblies grow in size and reach their maximum average size of ∼6.4 μm^2^ after 7 h (Figs. S11,†4f and g). This clearly confirms the effect of increasing ATP concentrations on the size and lifetime of the co-assembled structures.

**Fig. 5:**
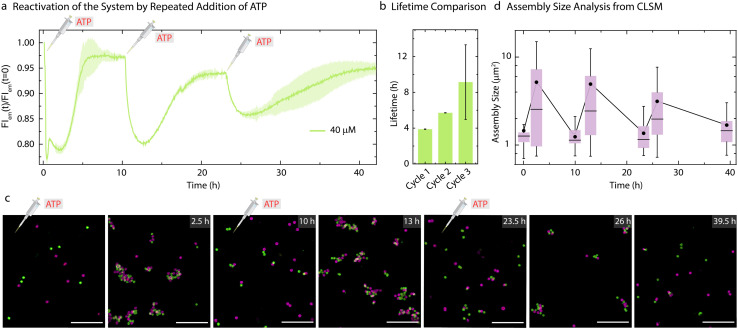
Reactivation of the system by repeated addition of ATP. (a) Time-dependent FI changes demonstrating transient co-assembly of dye functionalized Particle1-A1-Atto488 and quencher functionalized Particle2-A2-BMNQ535 upon repeated ATP addition. (b) Corresponding lifetimes for each consecutive cycle obtained from (a). Experimental conditions: Particle1-A1-Atto488 and Particle2-A2-BMNQ535 are suspended as an equimolar mixture in 1× NEB CutSmart buffer at a final MG concentration of 0.05 wt% containing 20 μM Complex 1, 5 μM Substrate 1, 10 μM Input 1 and Input 2 at 37 °C, fueled by repetitive addition of 40 μM ATP. The results represent an average contribution from two measurements, the shaded region in (a) and error bars in (b) depict the SD. (c) *Ex situ* CLSM imaging of transient co-assemblies between Particle1-A1-Atto488 and Particle2-A2-Atto647 at 40 μM ATP concentration. All CLSM images are represented as merged composite compiled as a *z*-projection. Experimental conditions: same as described in (a and b) except using Particle2-A2-Atto647 instead of Particle2-BMNQ535. Aliquots were withdrawn as needed and visualized without any dilution. (d) Assembly size analysis on the particles obtained from two different *z*-stacks at each time interval. The box represents 25–75% of data, whiskers represent 5–95% of data, a solid circle represents the mean, and horizontal bar the median of the assembly size distribution in box charts. A solid line provides a guide to the eye. Scale bars: 10 μm.

As the present system returns back to the initial state after consumption of ATP, this offered us the opportunity to reactivate the system with a fresh batch of fuel. Thus, we attempted refueling the system with three batches of ATP addition (Fig. 5). The time-dependent FI measurements (Fig. 5a) and CLSM analysis (Fig. 5c) demonstrate successful formation of co-assemblies between the particles which dissolve over time for each cycle. A quantitative analysis of the lifetimes using the time-dependent FI changes show an increase for subsequent fueling cycles (Fig. 5a and b). This is in line with the observations made for the pure ERN (Fig. S4†) and can be attributed to some loss of enzyme activities. Moreover, FI decreases by only 10% for the third cycle as compared to a 20% decrease for the first two cycles. This decrease in yield of the Assembly Complex associated with the transient co-assemblies is also reflected in the decreased size of the co-assemblies in the final cycle (Fig. 5c). The mean assembly size increases by only ∼2.3 times after the third batch of ATP addition as opposed to ∼4 times for the first two cycles (Fig. 5).

## Conclusion

In conclusion, we have demonstrated a generic design strategy for programming self-regulating co-assemblies of micron-sized particles when coupled to an ATP-driven ERN. Transient release of an Output strand from the upstream ERN acts as a linker for a pair of potentially co-assembling particles that indirectly pushes the co-assembled structures away from equilibrium. The key component is realized to be the length and composition of Output strand. It must be short enough to be easily ejected by the dual inversion strategy, but at the same time must have higher binding strength to bring two particles together. To achieve this, Output was strategically extended with a short domain (2 nt) which is only complementary to the co-assembling units but does not affect the release rate from the upstream ERN. This extra domain can be customized depending on the building blocks required to be programmed.

The system's properties can be tuned by simply changing the ATP concentration. For example, higher ATP concentration forces ligation to dominate restriction for longer periods which populates more Output strands in the dynamic steady state assuring larger co-assemblies to be sustained for longer periods. The second advantage stems from the resetting ability of the ERN because of which system can be successfully reactivated for at least three subsequent batches of ATP addition. The only waste accumulated in the system is the equivalent amount of AMP and PPi generated from the ATP used. Adding 40 μM of ATP accumulates 120 μM of AMP and PPi over three cycles which is well below the limit above which these components can affect MG or enzyme stability.^48,49^ Such conditions further favor the reactivation of the system.

Since the length of the linker can be further adjusted according to the strategy presented in this work, the system can be generalized for any building block based on DNA hybridization to produce materials with dynamic and tunable properties which can be sustained over multiple cycles. The presented colloidal particles can be grafted with biologically active molecules or ligands for communication with living cells opening up avenues for biomedical applications of fuel-driven matter.^40,50^

## Author contributions

Conceptualization, C. S., A. S., and A. W.; methodology, C. S. and A. S.; free solution studies, C. S. and A. S.; DNA synthesis and particle studies, C. S.; writing-original draft, C. S.; writing-review and editing, C. S., A. S., and A. W.; funding acquisition, A. W.; resources, A. W.; supervision, A. W.

## Conflicts of interest

There are no conflicts to declare.

## Supplementary Material

SC-014-D3SC04017H-s001

## Data Availability

All experimental and detailed procedures supporting this article have been uploaded as part of the ESI.†
