# Supplementary material for: Transient co-assemblies of micron-scale colloids regulated by ATP-fueled reaction networks
Source: Chem Sci. 2023 Oct 16;14(43):12299–307. doi: 10.1039/d3sc04017h (PMC10631234; doi:10.1039/d3sc04017h)
Supplement: SC-014-D3SC04017H-s001 [file SC-014-D3SC04017H-s001.pdf]

Electronic Supplementary Material (ESI) for Chemical Science.
This journal is © The Royal Society of Chemistry 2023

Supporting Information

Transient Co-Assemblies of Micro-Scale Colloids Regulated by ATP-Fueled Reaction Networks

Charu Sharma, Aritra Sarkar, Andreas Walther*

Life-Like Materials and Systems, Department of Chemistry, University of Mainz, Duesbergweg 10-14, 55128 Mainz, Germany.

andreas.walther@uni-mainz.de

Contents

1 Materials ____ *3*

2 General Characterization Methods and Instruments ____ *3*

3 Methods ____ *3*

3.1 Synthesis of surfactant-free, poly(2,2,2-trifluoroethyl methacrylate)-Rhodamine labeled core particles. ____ 3

3.2 Synthesis of the PNIPAM shell to yield PtFMA-Rhodamine labelled-core-PNIPAM-co-Acrylic Acid-shell microgel (MGs) particles. ____ 4

3.3 Functionalization of MG with amine-modified DNA. ____ 4

3.4 Synthesis and purification of amine-modified oligonucleotide sequences. ____ 4

3.5 DNA annealing ____ 5

3.6 CLSM image treatment and analysis ____ 5

3.7 Lifetime calculation ____ 5

4 Supplementary Notes ____ *5*

4.1 Definition of activity units of both enzymes ____ 5

5 Supplementary Tables ____ *6*

6 Supplementary Figures ____ *7*

7 References ____ *14*

1 Materials

All chemicals and reagents were purchased from Sigma Aldrich or Merck and used without further purification unless otherwise stated:

2,2,2-trifluoroethyl methacrylate (ABCR GMBH, 99 %), N-isopropylacrylamide (97 %), 2,2′-azobis(2-methylpropionamidine) dihydrochloride (ABCR GMBH, 96 %), N,N′-methylenebis(acrylamide) (99%), divinylbenzene (DVB, 80 %), acryloxyethyl thiocarbamoyl Rhodamine B (POLYSCIENCES, INC.), Al_2O_3 90 neutral (CARL ROTH GMBH), 1-Ethyl-3-(3-dimethylaminopropyl)carbodiimide (ABCR GMBH, 98 %), potassium persulfate (≥99 %), acrylic acid (Acros Organics, 99.5 %), disodium ethylenediaminetetraacetate dehydrate (biology grade), tris(hydroxymethyl)aminomethane hydrochloride (Trizma buffer substance pH=8), 2-(N-morpholino) ethanesulfonic acid, sodium chloride (99 %), magnesium chloride (99 %), phosphate buffered saline (PBS, pH = 7.4).

T4 DNA Ligase Storage Buffer (Promega): 10 mM Tris-HCl (pH 7.4 at 25 °C), 50 mM KCl, 1 mM dithiothreitol (DTT), 0.1 mM EDTA, 50 % glycerol. BsaI-HF® v2 storage buffer (*NEB*): 10 mM Tris-HCl, 200 mM NaCl, 1 mM DTT, 0.1 mM EDTA, 200 µg/mL BSA, 50% glycerol. 1X *NEB* CutSmart® Buffer: 50 mM potassium acetate, 20 mM Tris-acetate, 10 mM magnesium acetate, 100 µg mL-1 BSA. Annealing Buffer: 10 mM Tris-HCl (pH 8.0), 50 mM NaCl, 10 mM $MgCl_2$. MilliQ water was used throughout all experiments unless otherwise stated.

All oligonucleotides (except amine-modified oligonucleotides) were purchased from Integrated DNA Technologies Inc. (IDT) and Biomers GmbH (as listed below in Table S1). The oligonucleotides as received were dissolved in 1X TE buffer, pH = 8.0 (Thermo Fischer). Amine-modified oligonucleotides were synthesized following a well-defined procedure (as described in section 3.4).

2 General Characterization Methods and Instruments

DLS measurements were performed on the LS Instruments NanoLab 3D at 25 °C operating with a red laser (λ = 685 nm) and a scattering angle of ϴ = 90 ° using standard disposable PS cuvettes (BRAND GmbH & Co. KG). The distributions of the hydrodynamic radii were obtained by a CONTIN mode analysis.

Confocal laser scanning microscopy (CLSM) was performed on Leica Stellaris 5 microscope (LasX v4.3.0.24308) with four continuous wave laser lines (λ = 488 nm, power = 20 mW; λ = 561 nm, power = 20 mW; λ = 638 nm, power = 30 mW) and three HyD S detectors using plan-apochromat objectives (63×, 1.40 numerical aperture, oil immersion).

DNA concentrations were determined using a DeNovix-S-06873 (DeNovix OS 0.8.1 v4.1.5) spectrophotometer with a standard value of 33 µg/OD_{260}.

The temperature-controlled fluorescence measurements were performed on a TECAN (SPARK control v3.1) microplate plate reader using Corning® 384-Well black polystyrene plate with non-binding surface. Excitation and emission wavelengths for Atto488 are 485 nm and 535 nm and for Atto647N are 620 nm and 679 nm respectively.

***p* values** were calculated by performing a t-Test (Two-Sample Assuming Equal Variances) in Microsoft excel using a built-in Data analysis tool pack.

The amine-modified oligonucleotides were synthesized on a H-8 custom LNA, DNA/RNA automatic synthesizer from K&A Laborgeräte. The synthesized oligonucleotides were purified by **High Performance Liquid Chromatography (HPLC)** on a Dionex Ultimate 3000 (Thermo Fischer Scientific).

3 Methods

3.1 Synthesis of surfactant-free, poly(2,2,2-trifluoroethyl methacrylate)-Rhodamine labeled core particles.

The synthesis is analogous to a previous report.[1, 2] Divinylbenzene (DVB) and 2,2,2-trifluoroethyl methacrylate (tFMA) were purified using column chromatography (Al_2O_3, neutral). The initiator potassium persulfate (KPS,

159.49 mg, 590 µmol) was dissolved in deionized water (45 mL), degassed by bubbling with nitrogen gas (25 min) and thermostated at 70 °C for 15 min. To a solution of DVB (46.2 mg, 354.9 µmol) and tFMA (1.98 g, 11.8 mmol), a solution of acryloxyethyl thiocarbamoyl Rhodamine B (1 mg) and N-isopropylacrylamide (NIPAM, 150 mg, 1.3 mmol) in water (4.5 mL) was added. The mixture was ultrasonicated for 2 minutes and degassed by bubbling with nitrogen gas (10 min). The resulting mixture was added dropwise (over a period of 5 min) to the initiator solution starting the polymerization. The reaction mixture was stirred at 70 °C for 6 h (stirring rate = 600 rpm). The resulting dispersion was filtered while hot and dialyzed against deionized water (MWCO 8000 Da; solid content = 35.6 mg/mL by freeze-drying).

3.2 Synthesis of the PNIPAM shell to yield PtFMA-Rhodamine labelled-core-PNIPAM-co-Acrylic Acid-shell microgel (MGs) particles.

The synthesis was adapted from a previous report[1]: NIPAM (1.49 g, 13.2 mmol, 80.5 wt %) was dissolved in deionized water (100 mL) together with the cross-linker *N*,*N′*-methylenebis(acrylamide) (MBA, 18.2 mg, 81.7 µmol, 1.0 wt %). The core particles (4.7 mL, 35 mg/mL, 157.2 mg solid content, 9.5 wt %) were added and the mixture was degassed for 30 min and heated to 72 °C. The initiator KPS (76.2 mg, 0.28 mmol) was dissolved in water (30 mL) and degassed (15 min). The polymerization was initiated by dropwise addition of initiator solution to a heated reaction mixture and stirred (stirring rate = 350 rpm). After 10 minutes, degassed acrylic acid (AA, 153 mg, 150 µL, 2.1 mmol, 9 wt%) after purification by column chromatography (Al_2O_3, neutral) was added dropwise into the reaction mixture. The stirring rate was increased to 600 rpm and reaction was allowed to run for 4 hours. The resulting core/shell MG particles (amount of AA moieties assuming full conversion = 1250 µmol per g of MG) were filtered while hot and purified by dialysis (MWCO 8000 Da) against deionized water. The resulting core-shell MGs were further purified via centrifugation (5 × 25 min, 11000 rpm, 15 °C, replacement of the supernatant with Milli Q water per centrifugation step).

3.3 Functionalization of MG with amine-modified DNA.

The MG suspension was redispersed in 2-(N-morpholino)ethanesulfonic acid (MES) buffer (10 mM, pH = 4.5) via centrifugation (20 min, 9000 rpm, 15 °C, replacement of the supernatant with MES buffer). A two-step reaction achieves the functionalization of DNA on MGs. In the first step, activation of carboxyl acid groups in MG shell (120 µL, 1.943 mg/mL of MGs, 0.29 µmol of COOH groups) was carried out by stirring with EDC (1.1 mg, 7.2 µmol, 25 equiv. with respect to COOH groups) dissolved in 40 µL of MES buffer (10 mM MES, pH = 4.5) for 15 minutes at 25 °C. Finally, amine-modified DNA (0.1 µmol, 0.35 equiv.) dispersed in 350 µL PBS buffer (pH = 7.4) was added to activated MGs prepared in first step and the mixture was stirred for 4.5 h at 25 °C. The DNA functionalized MGs were purified via centrifugation (2 × 3 min, 8000 rpm, 25 °C, replacement of the supernatant with TE buffer (pH = 8.0), per centrifugation step).

3.4 Synthesis and purification of amine-modified oligonucleotide sequences.

The oligonucleotides were synthesized at 10 µmol scale employing the standard solid phase β-cyanoethyl-phosphoramidite chemistry in trityl-on mode. The DNA phosphoramidites (DMT (dimethoxytrityl)-dT, DMT-dA(bz), DMT-dG(dmf) and DMT-dC(ac)) were diluted to 50 mM with dry acetonitrile and synthesis occurred from the 3' towards the 5' end of the oligonucleotides on packed solid phase columns.

Cleavage of the oligonucleotides (DMT-on) from the solid support and base deprotection was achieved in one step to ensure optimal yields. The 34 µmol/g controlled pore glass (CPG) solid support was treated with 10 mL of ice-cold ammonia solution (30-32 % NH_3) overnight at room temperature to detach the DNA from the CPG support. The cleaved DNA (DMT-on) in ammonia was diluted with 10 mL of disodium phosphate buffer (75 mM containing 1 mM EDTA, pH = 8.3) and the crude product was obtained upon freeze drying. The obtained DNA was redispersed in MilliQ water and purified by preparative reverse phase-HPLC (RP-HPLC) followed by freeze drying to remove the solvent.

The DMT group was cleaved from the purified product by making a 2 wt % solution of the dry DNA in NaOAc/HOAc buffer (200 mM, pH 4.0, 200 mM NaCl) and heating the mixture to 50 °C for one hour. After neutralizing the reaction mixture with disodium phosphate buffer (750 mM, 10 mM EDTA), the synthesized DNA was precipitated into a 5-fold excess of isopropanol to remove contaminants and to exchange the counterions

to sodium. The precipitate was dissolved in MiliQ water and freeze dried. The synthesized and purified strands were stored at -20 °C until further use.

The purity of the obtained oligonucleotides was confirmed with analytical HPLC.

3.5 DNA annealing

All DNA strands were used as received. All the sequences are provided in Supplementary Table S1 and Table S2. The DNA strands received from IDT and Biomers were dissolved in TE buffer (10 mM Tris-HCl, pH = 8.0) to prepare a stock solution of 1 mM and stored at -20 °C for further use. The complementary DNA strands intended for double stranded complexes, i.e., Complex 1 and Substrate 1 were dissolved in annealing buffer (10 mM Tris-HCl, 50 mM NaCl, 10 mM $MgCl_2$, pH = 8.0) with the same stoichiometry at -20 °C overnight to prepare a stock solution of 0.125 mM.

3.6 CLSM image treatment and analysis

All CLSM images were processed using ImageJ (Fiji). All images except in Fig. 3a were acquired as a *z*-stack. Atto488, Atto647 and Rhodamine B channels were merged together to form a composite image and then compiled as a *z*-projection for representation purposes. The size of the co-assemblies was determined by thresholding the compiled images. The masks obtained upon thresholding were analyzed in terms of size and total amount.[3]

3.7 Lifetime calculation

The lifetime of the transient state is defined as the time that a transient profile (either obtained from time-dependent fluorescence measurements or assembly size analysis) takes to decrease to half of the initial and maximum value.

4 Supplementary Notes

4.1 Definition of activity units of both enzymes

Definition of the Weiss Unit to describe the activity of T4 DNA ligase: 0.01 Weiss Unit [WU] of T4 DNA Ligase is the amount of enzyme required to catalyze the ligation of greater than 95 % of 1 µg of λ/HindIII fragments at 16 °C in 20 minutes.

Unit definition to describe the activity of BsaI: One Unit [U] is defined as the amount of enzyme required to completely digest 1 µg of pXba DNA in one hour at 37 °C in 50 µl assay buffer containing acetylated BSA added to a final concentration of 0.1 g/L.

5 Supplementary Tables

Table S1. DNA sequences for the oligonucleotides synthesized following a well-defined procedure (section 3.4) with their abbreviations and modifications.

Name	Sequence 5'→ 3'	Figure	Modification
NH2-x* (T_{20}-x*)	TTTTTTTTTTTTTTTTTTTTT GAACCCGTATATCTATCCTA	Fig. 3-4	5' Amino Modifier C6 dT
NH2-z* (z*-T_{20})	GATAGAGATCGTGTGTTAC TTTTTTTTTTTTTTTTTTTTT	Fig. 3-4	3' Amino Modifier C6

Table S2. DNA sequences for the oligonucleotides with their abbreviations, sequence, and modifications. 5' Phosphorylated DNA strands were purchased from IDT. All other DNA sequences were purchased from Biomers.

Name	Sequence 5'→ 3'	Figure	Modification
Substrate	CGGATTGGTATTGTATTACC	Fig. 2-4	None
	AATCTTTAATACAATACCAATCCGATT	Fig. 2-4	5' Phos
Complex	CATGAGAATTCCATTCACGGTCTCT	Fig. 2-4	None
	GATTAGAGACCGTGAATGGAATTCTCATG	Fig. 2-4	5' Phos
Input 1	AATCAATCGGA	Fig. 2-4	5' Phos
Input 2	GTATTAAA	Fig. 2-4	None
A1-Atto488	ACCAATCCG TTTTTTTTTTTTTTTTTTTTT	Fig. 2	5' ATTO 488
A2-Atto647	TTTTTTTTTTTTTTTTTTTTT GGTAATACAAT	Fig. 2	3' BMN Q535
x-A1-Atto488	ACCAATCCG TAGGATAGATATACGGGTTC	Fig. 3,4	5' ATTO 488
z-A2-BMNQ535	GTAACACACGATCTCTATC GGTAATACAAT	Fig. 4a,4b,4c	3' BMN Q535
z-A2-Atto647	GTAACACACGATCTCTATC GGTAATACAAT	Fig. 3,4d,4e,4f,4g	3' ATTO 647N

6 Supplementary Figures

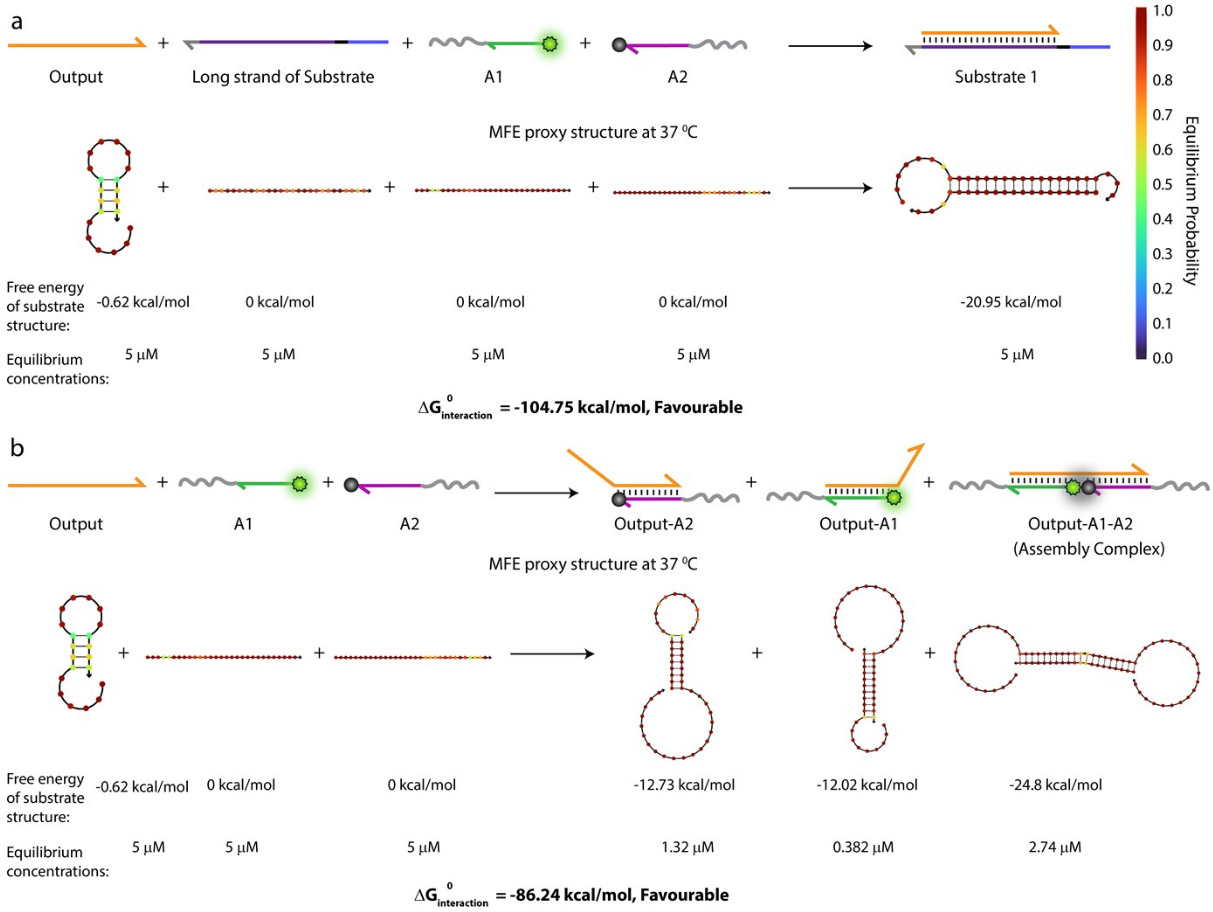

Fig. S1 Free energy change for the interaction between (a) Output and long strand of Substrate in the presence of A1 and A2 in the State A of the system, (b) Output and A1 and A2 in the absence of long strand of Substrate 1 in the State B of the system calculated with NUPACK simulations setting the temperature at 37 °C and salt concentrations at 50 mM NaCl, 10 mM $MgCl_2$. Output preferably remains bound within Substrate 1 and does not show any interaction with A1 or A2 in State A of the system. Only in State B of the system in the absence of long strand of Substrate, Output forms Assembly Complex with A1 and A2.

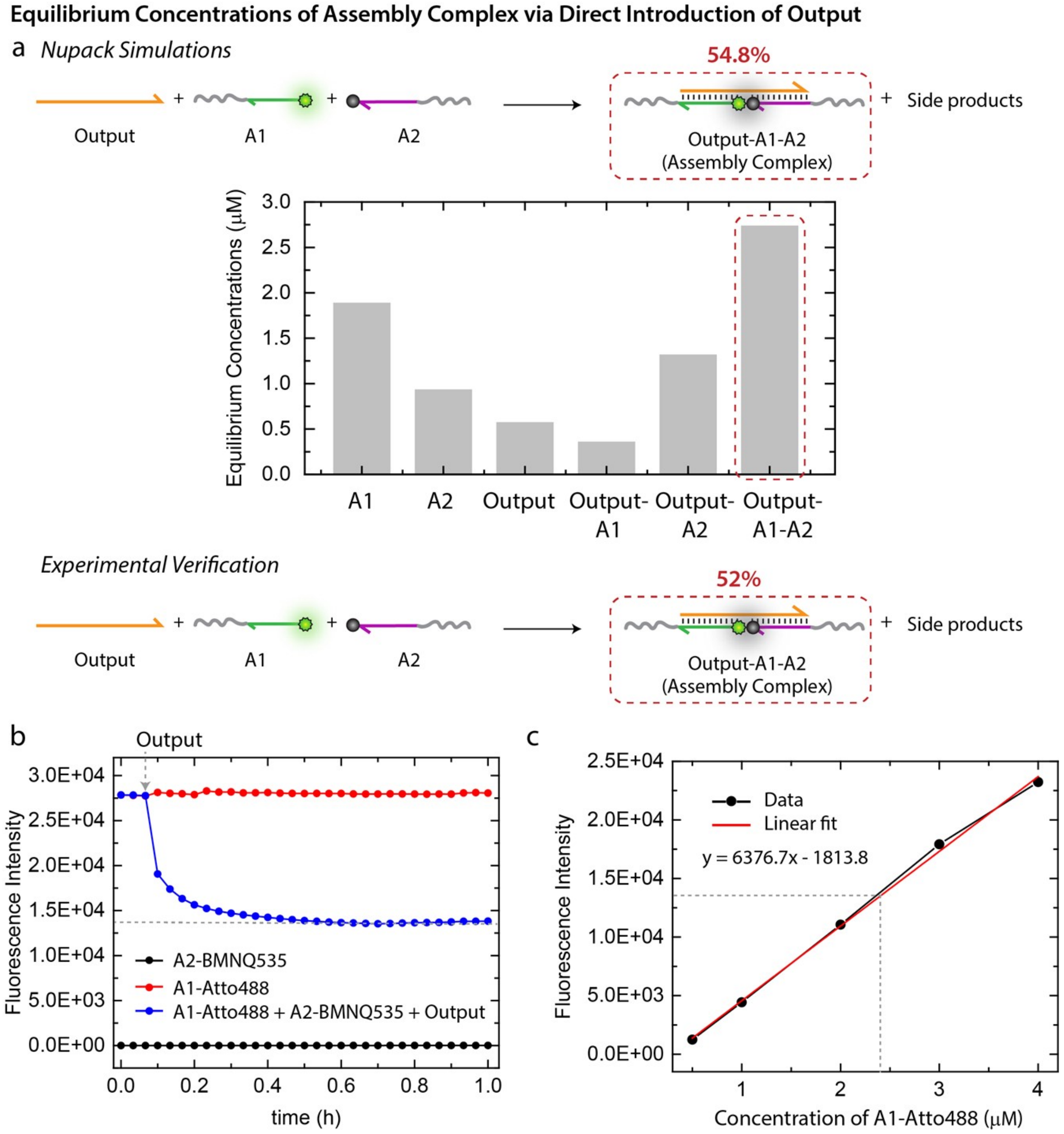

Fig. S2 (a) Equilibrium concentrations of all the components obtained from NUPACK[4] simulations upon mixing 5 µM each of Output, A1 and A2 setting the temperature at 37 °C and salt concentrations at 50 mM NaCl, 10 mM $MgCl_2$. The desired Assembly Complex between Output, A1 and A2 is formed in 54.8 % yield. (b), (c) Experimental verification of the equilibrium concentration of desired Assembly Complex between Output, A1-Atto488 and A2-ABMNQ535. (b) Time-dependent fluorescence intensity measurements of A1-Atto488 (red), A2-ABMNQ535 (black), and a 1:1 mixture of A1-Atto488, A2-ABMNQ535 upon introduction of Output in the system (blue). The remaining fluorescence (blue) corresponds to the uncomplexed or free A1-Atto488. (c) With the help of calibration curve between fluorescence Intensity and concentration of free A1-Atto488, the amount of uncomplexed A1-Atto488 in the equivalent mixture of A1-Atto488, A2-ABMNQ535, and Output can be calculated as 2.4 µM. Assuming a 100% quenching efficiency between Atto488 and BMNQ535 and zero fluorescence contribution from BMNQ535, the equilibrium concentration of Assembly complex can be calculated as 2.6 µM accounting for 52% yield. This means the experimental yield shows only 5% deviation from simulated yield (NUPACK, 54.8%). Experimental conditions: all species present at 5 µM concentration in 1X *NEB* CutSmart buffer at 37 °C.

Static and Transient Concentrations of Assembly Complex via Ligation Induced Release of Output

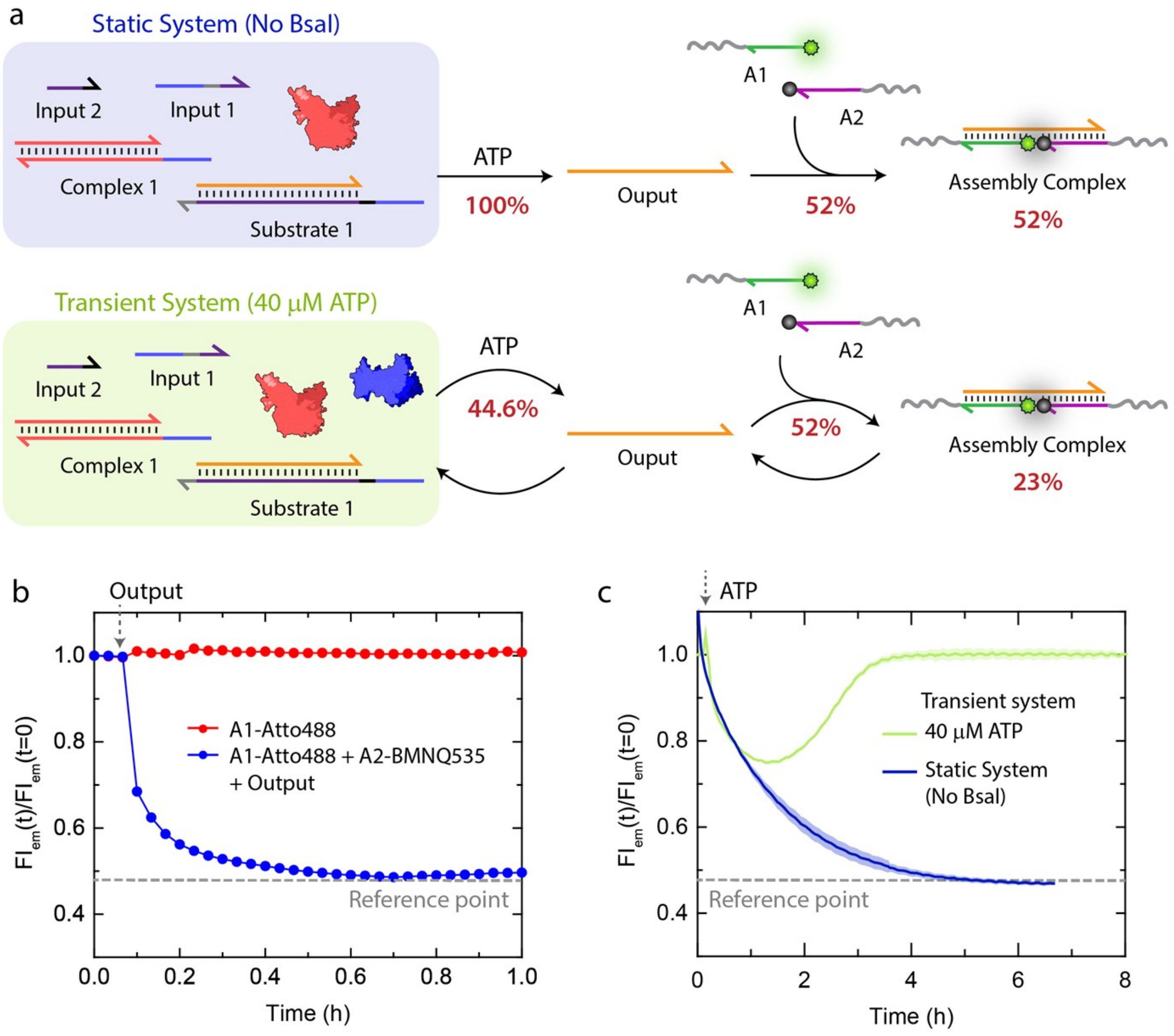

Fig. S3 (a) Schematics for experimental verification of percentage yield of Assembly Complex in Static and Transient systems. (b) Time-dependent fluorescence intensity measurements of A1-Atto488 and a 1:1 mixture of A1-Atto488, A2-ABMNQ535 upon introduction of Output in the system. The percentage of fluorescence decrease (grey, dotted line) provides a Reference point which indicates the minimum fluorescence that can be observed in the system. Experimental conditions: all species present at 5 µM concentration in 1X *NEB* CutSmart buffer at 37 °C. (c) Time-dependent fluorescence intensity changes demonstrating static and transient complexation of A1-Atto488 and A2-BMNQ535 upon ATP-fueled ligation induced release of Output. For the Static system, in the absence of BsaI, fluorescence intensity decreases approximately to the Reference point. This clearly indicates that Output must have released with 100% efficiency. But, in case of transient system, restriction can set in already at the hemi-ligated intermediate (e.g., Substrate 1, Complex 1 and only one of the Inputs) without completing to the fully ligated state which is the condition for expulsion of Output. Because of this, fluorescence intensity decreases by only 25% which indicates that Output is released with 44.6% efficiency with respect to Reference point. Since only 52% of Output released can form Assembly Complex (Fig. S2b), a final yield of 23% can be attributed to Assembly Complex. Experimental conditions: A1-Atto488 and A2-BMNQ535 at an equimolar concentration of 5 µM are dissolved in 1X *NEB* CutSmart buffer containing 20 µM Complex 1, 5 µM Substrate 1, 10 µM Input 1 and Input 2 at 37 °C. For transient system (green curve), 0.8 WU μL^{-1} of T4 DNA ligase and 0.8 U μL^{-1} of BsaI was used and system was fueled by 40 µM ATP; static system (blue curve) was carried out only with 0.8 WU μL^{-1} of T4 DNA ligase and fueled with 40 µM ATP.

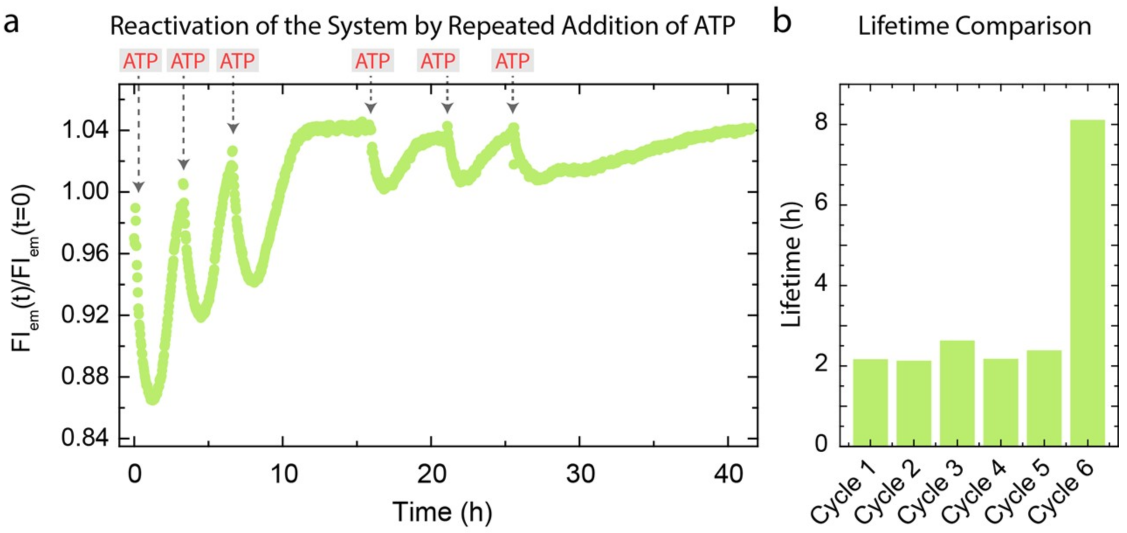

Fig. S4 Multiple activations of the system by repetitive addition of ATP. (a) Time-dependent fluorescence intensity changes upon three times addition of 40 µM ATP. (b) Corresponding lifetimes for each consecutive cycle obtained from Fig. S4a. Experimental conditions: A1-Atto488 and A2-BMNQ535 at an equimolar concentration of 5 µM are dissolved in 1X *NEB* CutSmart buffer containing 20 µM Complex 1, 5 µM Substrate 1, 10 µM Input 1, Input 2, 0.8 WU µL^{-1} T4 DNA ligase, and 0.8 U µL^{-1} BsaI at 37 °C, initiated by repetitive addition of 40 µM ATP.

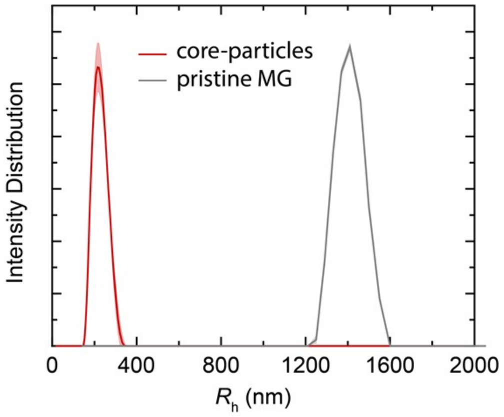

Fig. S5 DLS CONTIN plots of: poly(2,2,2-trifluoroethyl methacrylate) (PtFMA) core-particles and pristine MG. The suspensions were prepared in MilliQ water with a final concentration of 0.06 mg/mL.

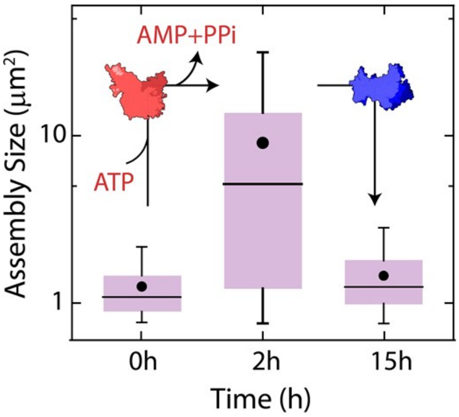

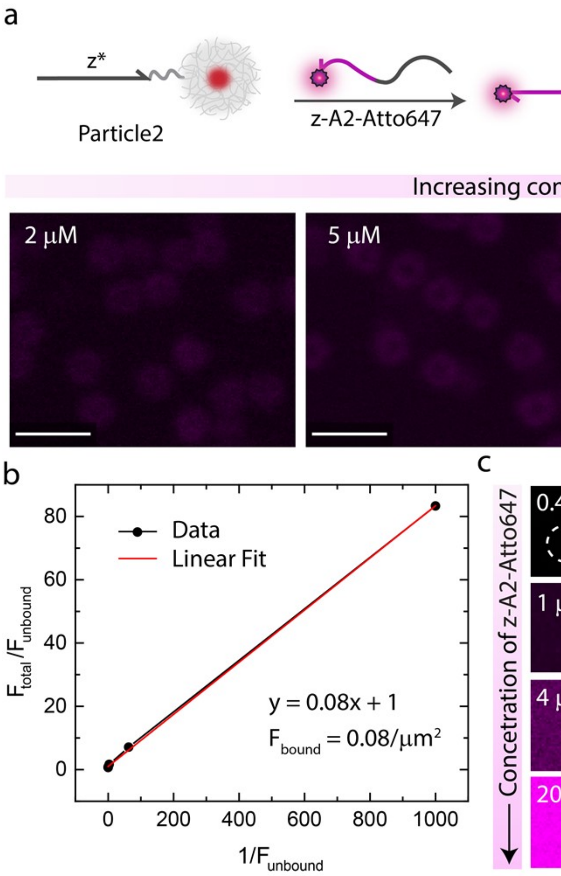

Fig. S6 Measurement of DNA grafting density on Particle2. (a) The binding capacity of z-A2-Atto647 onto Particle2 via z/z* hybridization is measured as a proxy for the DNA grafting density. The mean fluorescence intensity per μm^2 over the particle (F_{total}, includes the contribution from both z-A2-Atto647 bound on Particle2 and free z-A2-Atto647 in the suspension) and in the background ($F_{unbound}$, includes only free z-A2-Atto647 in the suspension) is measured for increasing amounts of z-A2-Atto647 via CLSM. Experimental conditions: Particle1 is incubated with increasing concentrations of z-A2-Atto647 (0.2-20 μM) in TE buffer (pH = 8.0) at 15 °C at a final MG concentration of 0.05 wt %. F_{total} and $F_{unbound}$ represent average fluorescence intensity measured from 10 different regions. (b) The data is fitted with linear equation where the slope provides fluorescence contribution from z-A2-Atto647 on the particle (F_{bound}). (c) With the help of calibration curve between mean fluorescence intensity per μm^2 (F, measured via CLSM) and concentration of free z-A2-Atto647, a corresponding DNA concentration for F_{bound} is calculated to be 4.5 ± 0.1 μM accounting for 2.7 × 10^3 strands/MG. Experimental conditions: Increasing concentrations of z-A2-Atto647 (4-40 μM) dispersed in TE buffer. F represents an average fluorescence intensity from 5 different regions. A laser with λ = 638 nm and power of 30 mW was used at 2% intensity throughout the experiment. Scale bars: (a), (c) 2 μm.

Fig. S7 Assembly size analysis on the particles from the CLSM images (Fig. 3b) obtained during sequential ATP fueling and BsaI restriction (i.e., static system) The co-assemblies achieve a maximum average size of 9 µm^2 after 2h of ATP addition. The formed structures disassemble again after 15h of BsaI addition. The box represents 25-75 % of data, whiskers represent 5-95 % of data, a solid circle represents the mean, and horizontal bar the median of the assembly size distribution in box charts.

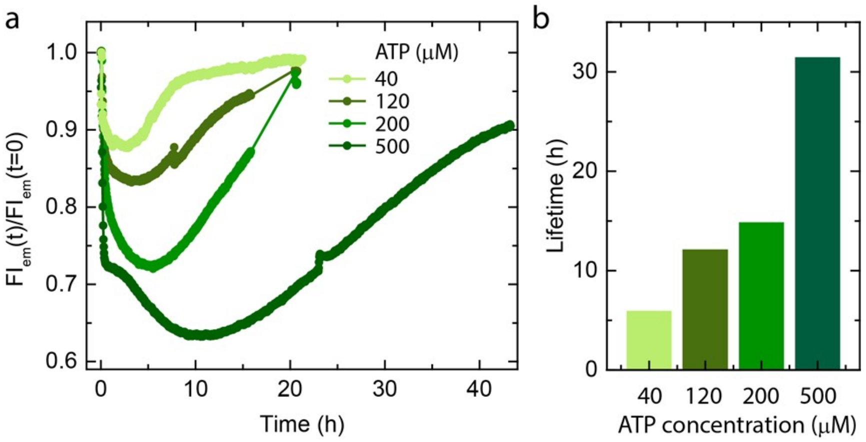

Fig. S8 (a) Time-dependent fluorescence intensity changes demonstrating transient co-assembly of dye-functionalized Particle1-A1-Atto488 and quencher-functionalized Particle2-A2-BMNQ535 at different ATP concentrations. (b) Lifetime comparison of the ATP-driven transient co-assembly of Particle1-A1-Atto488 and Particle2-A2-BMNQ535 with increasing ATP equivalents demonstrate consequent increase in lifetime. Experimental conditions: Particle1-A1-Atto488 and Particle2-A2-BMNQ535 are suspended as an equimolar mixture in 1X *NEB* CutSmart buffer at a final MG concentration of 0.05 wt% containing 20 µM Complex 1, 5 µM Substrate 1, 10 µM Input 1 and Input 2 at 37 °C, fueled by different ATP concentrations.

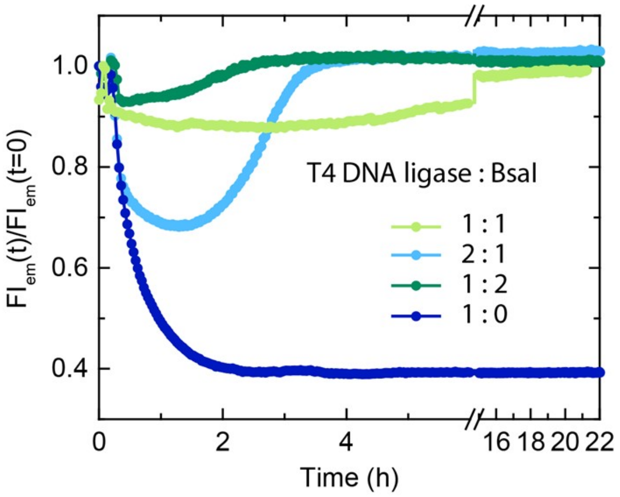

Fig. S9 Time-dependent fluorescence intensity changes demonstrating transient co-assembly of dye-functionalized Particle1-A1-Atto488 and quencher-functionalized Particle2-A2-BMNQ535 upon ATP addition at different ratios of T4 DNA ligase and BsaI. Please note that a 1:1 ratio of T4 DNA ligase and BsaI is used in all other experiments (standard conditions of the MS). Experimental conditions: Particle1-A1-Atto488 and Particle2-A2-BMNQ535 are suspended as an equimolar mixture in 1X *NEB* CutSmart buffer at a final MG concentration of 0.05 wt% containing 20 µM Complex 1, 5 µM Substrate 1, 10 µM Input 1 and Input 2 at 37 °C, fueled by 40 µM ATP.

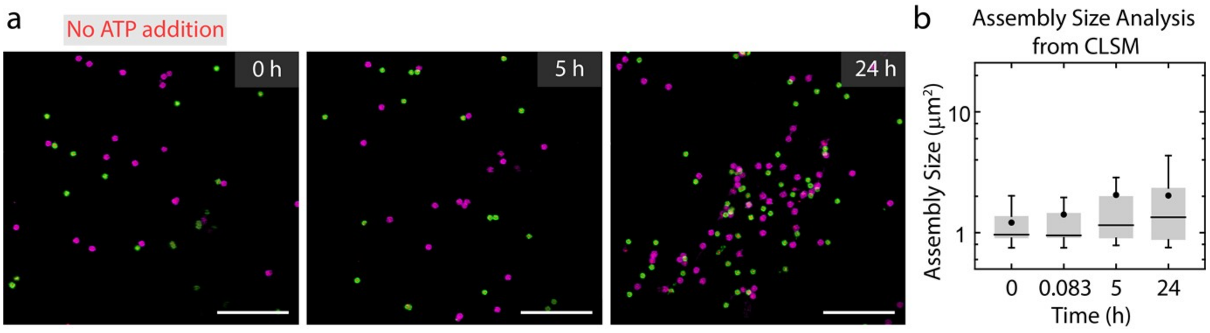

Fig. S10 Control for transient co-assemblies of MGs regulated by upstream ATP-fueled ERN without ATP addition. (a) Time dependent *ex situ* CLSM imaging shows absence of any co-assembly between Particle1-A1-Atto488 and Particle2-A2-Atto647 without ATP addition. All CLSM images are represented as merged composite compiled as a *z*-projection. Experimental conditions: Particle1-A1-Atto488 and Particle2-A2-Atto647 suspended as an equimolar mixture in 1X *NEB* CutSmart buffer at a final MG concentration of 0.05 wt %, 20 µM Complex 1, 5 µM Substrate 1, 10 µM Input 1 and Input 2 at 37 °C. (b) Assembly size analysis on the particles obtained from two different *z*-projections at each time interval. The average assembly size increased to 2.5 times of its initial value indicating formation of small clusters only after 24 h of monitoring the system. This might result because of salt-induced non-specific aggregation of MGs.[5] The box represents 25-75 % of data, whiskers represent 5-95 % of data, a solid circle represents the mean, and horizontal bar the median of the assembly size distribution in box charts. Scale bars: 10 µm.

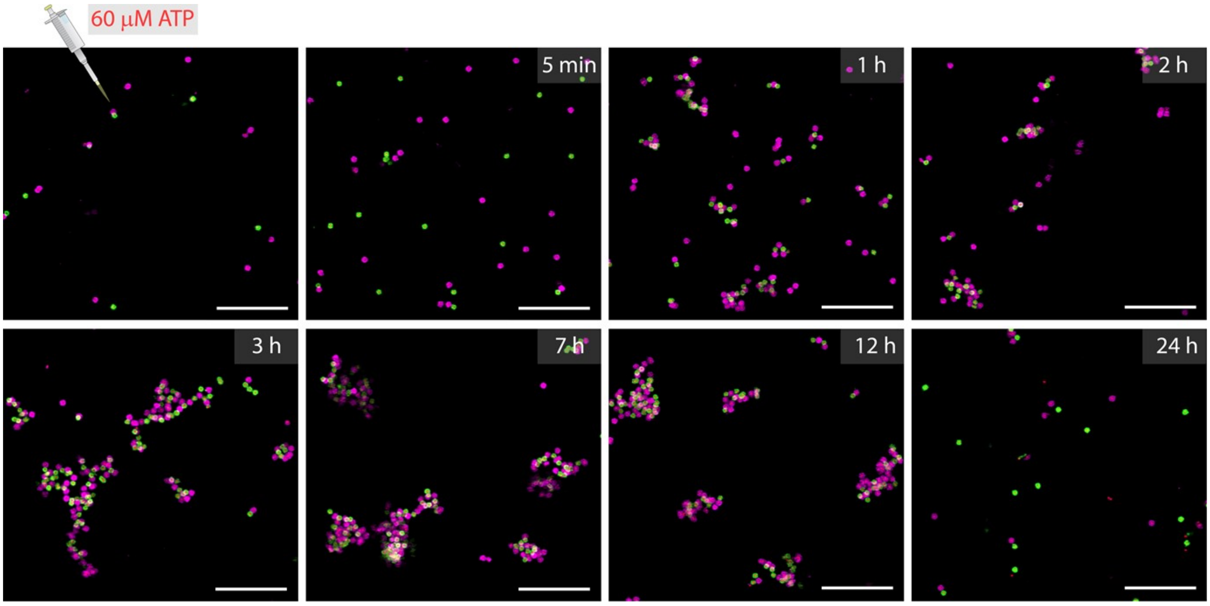

Fig. S11 Time dependent *ex situ* CLSM imaging shows transient co-assembly of MGs regulated by ATP-fueled upstream ERN. Experimental conditions: Particle1-A1-Atto488 and Particle2-A2-Atto647 suspended as an equimolar mixture in 1X CutSmart buffer at a final MG concentration of 0.05 wt%, 20 µM Complex 1, 5 µM Substrate 1, 10 µM Input 1 and Input 2 fueled by 60 µM of ATP at 37 °C. All CLSM images are represented as merged composite compiled as a *z*-projection. Scale bars: 10 µm.

7 References

1. K. Han, D. Go, D. Hoenders, A. J. C. Kuehne and A. Walther, *ACS Macro Lett.*, 2017, **6**, 310-314.
2. C. Sharma, A. Samanta, R. S. Schmidt and A. Walther, *J. Am. Chem. Soc.*, 2023, **145**, 17819-17830.
3. H. Dehne, A. Reitenbach and A. R. Bausch, *Sci. Rep.*, 2019, **9**, 7350.
4. J. N. Zadeh, C. D. Steenberg, J. S. Bois, B. R. Wolfe, M. B. Pierce, A. R. Khan, R. M. Dirks and N. A. Pierce, *J. Comput. Chem.*, 2011, **32**, 170-173.
5. A. F. Routh and B. Vincent, *Langmuir*, 2002, **18**, 5366-5369.
